# Patterns of structure-function association in normal aging and in Alzheimer's disease: Screening for mild cognitive impairment and dementia with ML regression and classification models

**DOI:** 10.3389/fnagi.2022.943566

**Published:** 2023-02-23

**Authors:** Yauhen Statsenko, Sarah Meribout, Tetiana Habuza, Taleb M. Almansoori, Klaus Neidl-Van Gorkom, Juri G. Gelovani, Milos Ljubisavljevic

**Affiliations:** ^1^Department of Radiology, College of Medicine and Health Sciences, United Arab Emirates University, Al Ain, United Arab Emirates; ^2^Big Data Analytics Center (BIDAC), United Arab Emirates University, Al Ain, United Arab Emirates; ^3^Department of Medicine, University of Constantine 3, Constantine, Algeria; ^4^College of Information Technology, United Arab Emirates University, Al Ain, United Arab Emirates; ^5^Department of Surgery, College of Medicine and Health Sciences, United Arab Emirates University, Al Ain, United Arab Emirates; ^6^Biomedical Engineering Department, College of Engineering, Wayne State University, Detroit, MI, United States; ^7^Siriraj Hospital, Mahidol University, Salaya, Thailand; ^8^Department of Physiology, College of Medicine and Health Sciences, United Arab Emirates University, Al Ain, United Arab Emirates; ^9^Abu Dhabi Precision Medicine Virtual Research Institute (ADPMVRI), United Arab Emirates University, Al Ain, United Arab Emirates

**Keywords:** brain morphometry, structural-functional association, artificial intelligence, neurophysiological test, cognitive score, aging, cognitive decline, Alzheimer's disease

## Abstract

**Background:**

The combined analysis of imaging and functional modalities is supposed to improve diagnostics of neurodegenerative diseases with advanced data science techniques.

**Objective:**

To get an insight into normal and accelerated brain aging by developing the machine learning models that predict individual performance in neuropsychological and cognitive tests from brain MRI. With these models we endeavor to look for patterns of brain structure-function association (SFA) indicative of mild cognitive impairment (MCI) and Alzheimer's dementia.

**Materials and methods:**

We explored the age-related variability of cognitive and neuropsychological test scores in normal and accelerated aging and constructed regression models predicting functional performance in cognitive tests from brain radiomics data. The models were trained on the three study cohorts from ADNI dataset—cognitively normal individuals, patients with MCI or dementia—separately. We also looked for significant correlations between cortical parcellation volumes and test scores in the cohorts to investigate neuroanatomical differences in relation to cognitive status. Finally, we worked out an approach for the classification of the examinees according to the pattern of structure-function associations into the cohorts of the cognitively normal elderly and patients with MCI or dementia.

**Results:**

In the healthy population, the global cognitive functioning slightly changes with age. It also remains stable across the disease course in the majority of cases. In healthy adults and patients with MCI or dementia, the trendlines of performance in digit symbol substitution test and trail making test converge at the approximated point of 100 years of age. According to the SFA pattern, we distinguish three cohorts: the cognitively normal elderly, patients with MCI, and dementia. The highest accuracy is achieved with the model trained to predict the mini-mental state examination score from voxel-based morphometry data. The application of the majority voting technique to models predicting results in cognitive tests improved the classification performance up to 91.95% true positive rate for healthy participants, 86.21%—for MCI and 80.18%—for dementia cases.

**Conclusion:**

The machine learning model, when trained on the cases of this of that group, describes a disease-specific SFA pattern. The pattern serves as a “stamp” of the disease reflected by the model.

## 1. Introduction

Studies in cognitive neuroscience aim to explain the operation of the human mind (Thagard, 2013). For this, researchers construct different models predicting brain functioning. The models provide a potential explanation of how the brain processes information although not all of them implement neuron-like elements (Forstmann and Wagenmakers, 2015; Palmeri et al., 2017). The research questions and the study design determine the conceptual architecture of the models (Beer, 2000). The purpose of our research is to distinguish normal aging from the accelerated one which manifests itself with dementia. We also aim to improve the early-stage diagnostics of mild cognitive impairment (MCI). The multimodal diagnostics seems to be one of the most promising ways to reach the objective. It is more sensitive for identification and prognosis of Alzheimer's disease (AD). Therefore, this technique is suitable for screening and designing early management strategies (Perrin et al., 2009). However, the concept of multimodality is not clearly defined. In some references it denotes the combined analysis of distinct structural MRI sequences (Kang et al., 2020). However, the comprehensive analysis of the brain structure is more informative when the data on the brain metabolism are considered (Yu et al., 2012; Willette et al., 2014; Forouzannezhad et al., 2018; Lin et al., 2020). The invasiveness and high cost of the metabolic studies make them non-applicable for screening purposes. Contrarily, a combination of structural and functional data seems to be a reasonable alternative: it is easy to conduct, more affordable, and reliable (Sabbagh et al., 2017). Cognitive examination can improve the diagnostic and predictive power of routine clinical investigations such as MRI (Willette et al., 2014; Ottoy et al., 2019; Lin et al., 2020), SPECT (Borroni et al., 2006; Quaranta et al., 2018), and PET (Yu et al., 2012; Teng et al., 2020). A combined study of structural and functional data may provide understanding of the AD pathogenesis. The multimodal diagnostics should benefit from the advantages of various methods and overcome their disadvantages. Such an approach continues to be a subject of the ongoing research since the existing models of multimodal screening have a set of limitations, such as complex feature engineering (Lu et al., 2018; Kang et al., 2020), low accuracy (Quaranta et al., 2018), and low practical value due to the small sample size. These weaknesses limit translating the models into clinics (Drzezga et al., 2005; Zhang et al., 2011; Yu et al., 2012). Meanwhile, our idea is to focus on the change both in the brain structure and function in normal and accelerated aging. We suppose to overcome the aforementioned limitations by proposing new multimodal diagnostic models. To use the advances of the multimodal diagnostics, we resort to structure-function association (SFA) models that can justify distinct SFA patterns in healthy individuals, patients with MCI and AD (Habuza et al., 2021a,b,c,d,e, 2022). The practical value of these findings is promising: they can be used to construct multimodal diagnostic machine learning (ML) algorithms.

## 2. Objectives

We intend to get an insight into normal and accelerated brain aging by developing ML models to predict individual performance in neuropsychological and cognitive tests from brain MRI. With these models we endeavor to look for brain SFA patterns indicative of MCI and Alzheimer's dementia. We suppose that the combined analysis of imaging and functional modalities will improve diagnostics of neurodegenerative diseases with advanced data science techniques.

We will compare the diagnostic images and the results of neuropsychological and cognitive assessment with ML and deep learning (DL) techniques to determine if there are distinct patterns of age- and diseases-related change in brain morphometry, functioning, and SFA. *Alternatively*, if there are no patterns of this kind, there should be a common mode of structural deterioration and cognitive decline for aging and pathology. In this case some threshold level indicative of the disease should be established. ML will allow us to distinguish normal aging from pathology with the help of a classification model. We address the following sub-objectives:

1. Study dynamics of performance in cognitive and neuropsychological tests in healthy individuals, patients with MCI and dementia.
2. Build models of brain structure-function associations in cognitively normal individuals, patients with MCI and dementia.
3. Work out an approach for classification of examinees according to SFA patterns into the cohorts of the cognitively normal elderly, patients with MCI and dementia.

## 3. Materials and methods

### 3.1. Study participants

For the study, we used data from a publicly available Alzheimer's Disease Neuroimaging Initiative database (ADNI).^1^ ADNI1 includes 400 subjects diagnosed with MCI, 200 subjects with early AD, and 200 elderly control subjects in the 55–90 age range (ADN, 2019, 2022). See inclusion and exclusion criteria in ADNI general procedures manual (ADNI General Procedures Manual, 2004). The ADNI was launched in 2003 as a public-private partnership, led by Principal Investigator Michael W. Weiner, MD. The primary goal of ADNI has been to test whether serial MRI, PET, other biological markers, and clinical and neuropsychological assessment can be combined to measure the progression of MCI and early AD. In this study, we acquired MRI and clinical information on all the cases collected to ADNI1 dataset in a cross-sectional and longitudinal study design. This provided us with a total number of 1,337 study cases from 800 subjects. We excluded 35 cases from our study because of a failure of FreeSurfer to segment the brain MRI. We also excluded all patients who changed the group (e.g., CN to MCI or dementia) so convertible cases did not affect the study results. Examinations of the same subjects in the same study group were carried out with a certain time interval. Logically, the results of these examinations reflected either the normal or the pathological class-specific SFA pattern during aging. The findings did not present the individuals at a common age or disease stage. In this way, there was no direct data leakage between training and testing datasets within a cohort. Same subjects in the same study group had no effect on test results when prediction was performed with unseen data from other classes. For the remaining 1,302 cases we collected demographic data, pre-processed T1-weighted MRI files and results in the following cognitive tests: the mini-mental state examination (MMSE), the Rey auditory verbal learning test (RAVLT), part B of the trail making test (TMT), the digit symbol substitution test (DSST), the Alzheimer's disease assessment scale-cognitive subscale (ADAS-cog). See Table 1 for the descriptive statistics on the study cohorts.

**Table 1:** Demographics, cognitive performance in study cohorts.

		**Total**	**Cognitively normal**	**MCI**	**Dementia**	**p_1__−__3_**
		***n* = 1,302**	***n*_1_=287 (22.04%)**	***n*_2_=646 (49.62%)**	***n*_3_=369 (28.34%)**	
**Years**
	Age	75.74 [71.7–80.7]	76.62 ± 5.62	75.25 ± 7.16	75.93 ± 7.37	0.0933785
	Education	15.58 [13.0–18.0]	16.13 ± 2.91	15.76 ± 2.99	14.85 ± 3.21*	**9.08991e-08**
**Sex**
	Women	522 (40.09%)	134 (46.69%)	215 (33.28%)	173 (46.88%)	**4.19707e-06**
	Men	780 (59.91%)	153 (53.31%)	431 (66.72%)	196 (53.12%)
**Race**
	Whites	1,210 (92.93%)	261 (90.94%)	603 (93.34%)	346 (93.77%)	0.198438
	Blacks	60 (4.61%)	21 (7.32%)	22 (3.41%)	17 (4.61%)		
	Asians	30 (2.3%)	5 (1.74%)	19 (2.94%)	6 (1.63%)		
**Test** **result**
	ADAS-cog	19.87 [11.7–26.3]	8.73 ± 4.14	18.82 ± 6.6	30.37 ± 8.97	**2.2404e-165**
	MMSE	26.18 [24.0–29.0]	29.06 ± 1.09	26.91 ± 2.2	22.66 ± 3.03	**2.1560e-155**
	RAVLT	30.44 [23.0–37.0]	43.2 ± 9.76	29.79 ± 8.86	21.67 ± 7.77	**3.7071e-120**
	DSST	36.24 [27.0–45.0]	46.77 ± 11.06	37.37 ± 11.1	26.05 ± 12.41	**2.72808e-83**
	TMT	138.13 [75.0–187.0]	85.03 ± 43.18	128.48 ± 72.56	200.96 ± 88.57	**2.20487e-73**

*p*−*value* is marked in bold if difference among groups is statistically significant (*p* < 0.05).

Statistical data are reported as *IQR*, *Mean*±*SD*, or absolute number of cases and their percentage in studied cohort.

If the distribution of metrics differs significantly (*p* < 0.05) for an index compared to other ones, its *Mean* ± *SD* is marked with an asterisk.

### 3.2. Brain morphometry and machine learning

To process T1-weighted brain images we used an open source toolkit FreeSurfer. In particular, we calculated subcortical and cortical parcellation volumes—voxel-based morphometry data (VBM) and surface-based morphometry data (SBM)—with FreeSurfer 7.1.0 software (Freesurfer Software Suite, 2011). As a reference we used Desikan-Killiany atlas. For each segmented area of the brain we calculated the absolute value as the average value between the right and left-side volume. Then we expressed the data in percentage to the total intracranial volume. The relative volumes of various brain areas were the predictors of the ML models which we trained to compute cognitive scores and results in psychophysiologic tests. The ML algorithms were trained on each study cohort separately: cognitively normal individuals (CN), patients with MCI and AD. Ranking the predictors by their informative values provided an insight into SFA patterns which can be either similar or different in these cohorts. If the SFA patterns differ among the studied groups, one can find the SFA model which describes the individual structural and functional data the best. This means, we can train an ML classification model to distinguish healthy aging from MCI or AD thus improving their early diagnostics. To combine the predictions from multiple contributing ensemble member models, we will use an ensemble ML technique called blending.

### 3.3. Cognitive and neuropsychological tests

The purely pre-symptomatic and early stages of dementia are likely to be identified by neuropsychological testing. The existing dementia risk models mainly comprise demographics, subjective cognitive complaints, lifestyle factors, health state estimates, and other variables (Hou et al., 2019). Cognitive test scores or neuropsychological test batteries are incorporated as predictors into many models of developing dementia. The most commonly used cognitive scores are MMSE (Folstein et al., 1983), RAVLT (Rey, 1964), ADAS-cog (Mohs, 1994), DSST (Wechsler, 1981), TMT (Spreen et al., 1998), Clinical Dementia Rating (Morris, 1997), Logical Memory Tests, Immediate and Delayed Recall (Wechsler, 1987).

We resorted to the following tests namely because of their availability in the ADNI1 dataset and also because of the ability to cover the global cognition (the first two tests) and some cognitive subdomains (the last three ones):

1. *ADAS-cognitive Subscale (ADAS-cog)* is an informative tool for monitoring neurodegeneration in clinical routine practice (Zhu et al., 2018). The test distinguishes between MCI and mild AD with sensitivity (Sn) of 0.86 and specificity (Sp)—0.89 (Zainal et al., 2016). It can also identify “questionable dementia” as its results in immediate recall and object naming tasks correlate with performance in Category Verbal Fluency Test (Lam et al., 2006).
2. *MMSE* is the most common method for diagnosing cognitive impairment in a single or multiple domains (Foley et al., 2018). Although it detects various types of dementia with high Sn and Sp (over 90%), the test should be accompanied by a full and detailed assessment of the patients (Fountoulakis et al., 2000). For this, clinicians use other tests (e.g., TMT, DSST) (Godefroy et al., 2011).
3. *TMT* provides information on neuropsychological conditions; therefore it is used for diagnosing neurodegenerative diseases in combination with other tests and diagnostic modalities (Godefroy et al., 2011; Ciulli et al., 2016; Olchik et al., 2017). Its clinical implication is multifold. First, TMT helps to define the impaired cognitive domain and improves the assessment with MMSE or Montreal Cognitive Assessment (MoCA) (Godefroy et al., 2011). Second, there is evidence that the inclusion of TMT-B boosts the performance of the models discriminating AD against non-AD MCI based on CSF and structural biomarkers (Ewers et al., 2012). Third, the test can sensitively distinguish a case of mild AD from amnestic MCI and healthy aging (Bottiroli et al., 2021).
4. *RAVLT* examines verbal learning and memory. It is capable of detecting cognitive impairment in multiple sclerosis (Beier et al., 2019). The test differentiates between AD dementia and behavioral variant of fronto-temporal dementia (Ricci et al., 2012) with high Sn and Sp (over 81%). It also helps physicians to distinguish AD from Lewy bodies dementia (Bussè et al., 2017).
5. *DSST* identifies early stages of dementia (Proust-Lima et al., 2007) and MCI by detecting working memory impairment and multimodal amnesia (Ciafone et al., 2021). The test also shows significantly impaired performance in early dementia with Lewy Bodies (Botzung et al., 2019).

The results in ADAS-cog and RAVLT are presented with a set of dependent variables: *ADAS*_*Q*4_, *ADAS*_11_, *ADAS*_13_ and *RAVLT*_*immediate*_, *RAVLT*_*learning*_, *RAVLT*_*forgetting*_ (ADN, 2022). In a recent study, we showed that the associations between CSF% and performance in ADAS-cog and RAVLT are stronger for ADAS-13 and *RAVLT*_*immediate*_ compared to the other scores in these tests (Habuza et al., 2022). For this reason we used ADAS-13 and *RAVLT*_*immediate*_ for further analysis.

### 3.4. Methodology of study

*To complete the first subobjective*, we assessed change of the cognitive and neuropsychological test scores in normal and accelerated aging. We explored the age-related variability of cognitive scores in the tests commonly used either to diagnose MCI and dementia or to improve the accuracy of multimodal diagnostics. The first group of the tests reflects the global cognitive functioning and it includes MMSE and ADAS-cog. The second group of the tests covers a few cognitive domains, i.e., information processing in DSST, memory in RAVLT, information processing in TMT. To present change in the test scores with the disease progression, we built distribution of the test results over age with linear trendlines and 95% confidence intervals for the study cohorts. Then we conducted comparative analysis of the three groups. We employed non-parametric statistical methods, i.e., a Chi-square test for the quantitative features and Kruskal-Wallis test for the continuous ones.

*To reach the second subobjective*, we constructed regression models predicting functional performance in cognitive tests from brain radiomics. We employed a ridge regression model with linear least squares function and L2-norm regularization. To control regularization strength, we set the parameter to the value of α = 0.5. The vulnerability of distinct neuronal cells to atrophy in accelerated aging differs among distinct cell groups and brain regions. Reasonably, SFA can have features specific to pathology. For checking this, we trained the regression models on the three study cohorts independently. The predictors of the model were the data acquired from voxel- and surface-based brain morphometry. The voxel-based morphometry is a computational approach to neuroanatomy that measures differences in local concentration of brain tissue *via* a voxel-wise comparison of multiple brain images. The surface-based brain morphometry is a complementary structural imaging analysis for quantifying gray matter abnormalities. The feature selection technique allowed us to identify the most valuable structural neuroimaging measures. We employed ensemble tree-based estimators such as Random Forest, and Extra Trees, AdaBoost, Gradient Boosting. These models were trained one by one on the data of the studied groups. We used impurity-based feature importance to rank the features according to their predictive potential. The models reflect SFA patterns specific for each study cohort. We also looked for significant correlations between cortical parcellation volumes and test scores in the cohorts to investigate neuroanatomical differences in the cognitive status.

**Figure 1:**
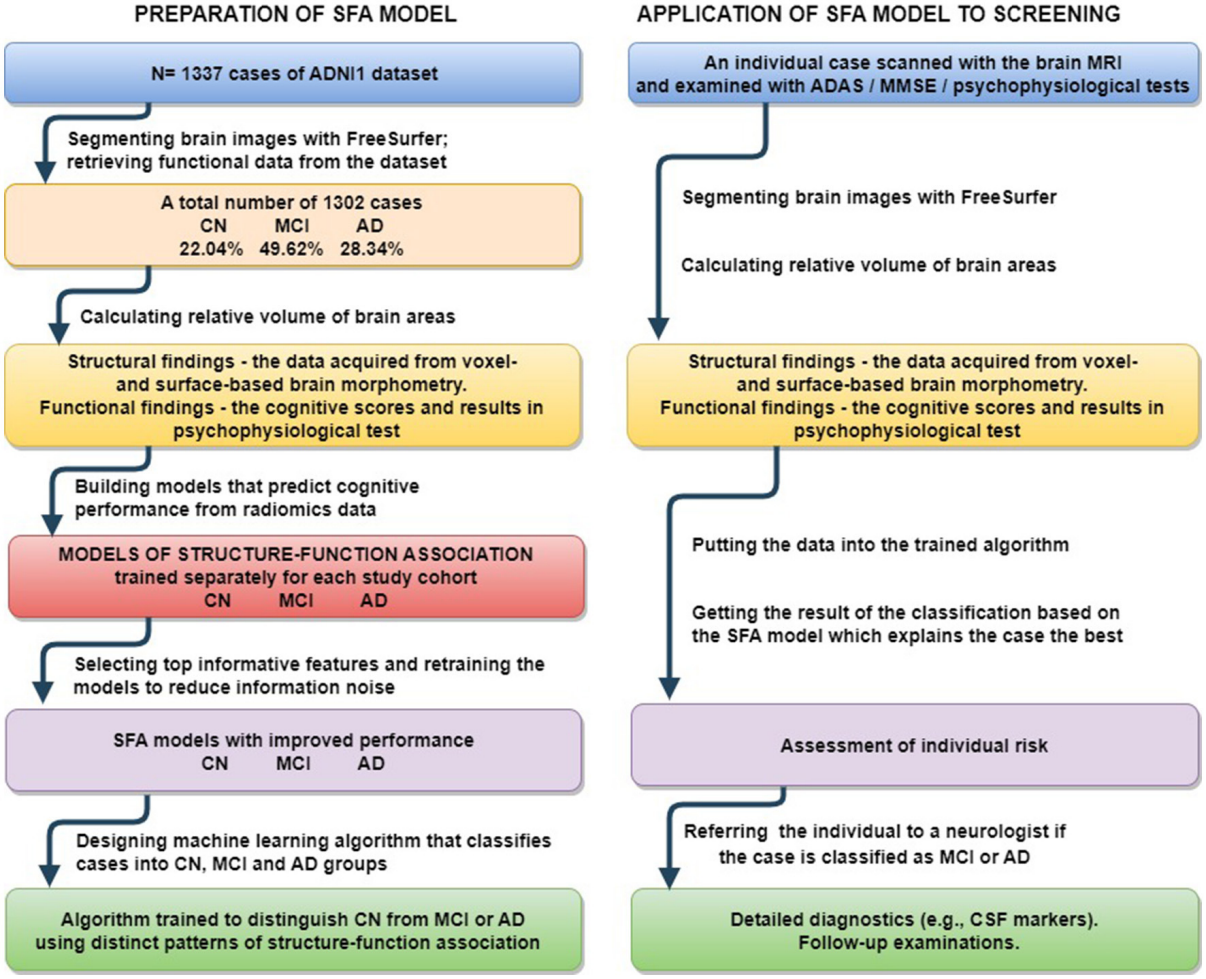
Preparation of proposed SFA classification model and its application to screening for early stage MCI and Alzheimer's disease.

*The third subobjective* was to assess the diagnostic value of the proposed models. We tried to classify individual findings according to the model which describes the case best (see Figure 1). The idea was that the ML model, when trained on the cases of this of that group, describes a disease-specific SFA pattern. The pattern serves as a “stamp” of the disease on which the model was trained. Therefore, one can find the “stamp” which fits the case best. We trained ridge regression models on groups of healthy people and patients diagnosed with MCI and dementia to predict cognitive test results. Then we calculated the difference between the predicted and actual test scores of the observed patient. The minimal difference identified the group. We followed these steps with results in MMSE, ADAS, RAVLT, DSST, and TMT. Afterwards we employed the majority voting technique to assess the performance of the multigroup classification.

### 3.5. Hardware and software

The calculations were done with the Linux Ubuntu 18.04 workstation with 24 CPU cores and two NVIDIA GeForce GTX 1080 Ti GPU with 11 GB GDDR5X memory each using programming language Python, and its libraries for Data Processing, ML, and Data visualization, such as scikit-learn, NumPy, Pandas, Matplotlib, and Seaborn.

## 4. Results

### 4.1. Performance in cognitive and neuropsychological tests in healthy individuals, patients with MCI and dementia

**Figure 2:**
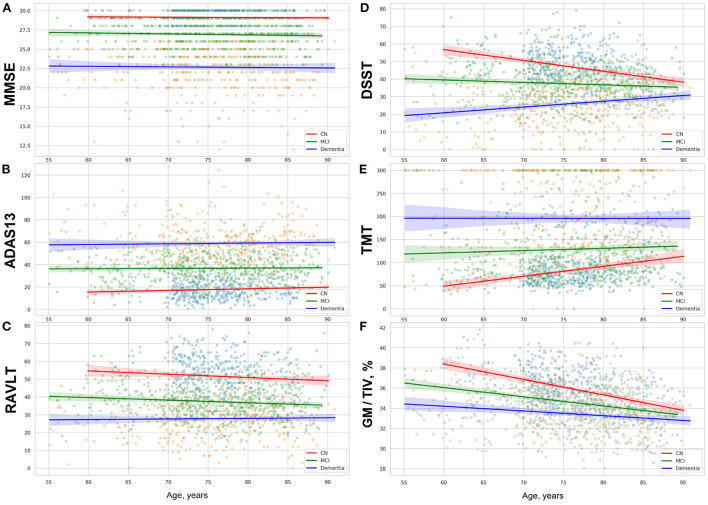
**(A)** MMSE, **(B)** ADAS13, **(C)** RAVLT scores, results in **(D)** DSST, **(E)** TMT, and **(F)** proportion of gray matter volume to total intracranial volume in group of cognitively normal adults and patients with MCI or dementia. The figures show linear trendlines with 95% confidence intervals.

To study how healthy individuals and patients with cognitive impairment perform, we carried out exploratory analysis of ADNI dataset. The test paradigm and the cognitive slowing due to normal or accelerated aging may influence individual scores. The functional data covering last decades of life, from 55 to 90's, show difference between the global cognition scores and results in test assessing a few cognitive subdomains. The global cognition ability measured with MMSE and ADAS13 remains stable with age in all the study cohorts (see Figures 2A,B). In contrast to this, we observe a steady decline in neuropsychological tests—DSST and TMT—with advancing age (see Figures 2D,E).

The rate of neurocognitive slowing is higher in cognitively preserved individuals compared to the patients with cognitive deterioration (see Table 2). If approximated to the age of over 100 years, the lines for the three cohorts will end up in a common point. The trends for performance in these tests resemble the dynamics of the gray matter atrophy (see Figure 2F). The results in RAVLT have age-related change roughly similar to the global cognitive scores but with a slight tendency toward convergence of the trends for the cognitively normal individuals, patients with MCI and those who suffer from AD.

**Table 2:** Interaction coefficients for comparison of slopes for cognitive performance and brain morphometry between CN, MCI, and Dementia groups [estimate ± SD, (CI)].

	**MMSE**	***p*-value**	**ADAS**	***p*-value**	**RAVLT**	***p*-value**	**TMT**	***p*-value**	**DSST**	***p*-value**	**GM/TIV**	***p*-value**
CN	−0.005 ± 0.021	0.827	0.092 ± 0.063	0.148	−0.174 ± 0.082	**0.035**	2.126 ± 0.649	**0.001**	−0.616 ± 0.104	**3.65e-09**	−0.153 ± 0.019	**9.24e-16**
	[−0.045; 0.036]		[−0.033; 0.216]		[−0.335; −0.012]		[0.854; 3.398]		[−0.820; −0.413]		[−0.190; −0.116]
CN vs.	−0.008 ± 0.024	0.73	−0.070 ± 0.074	0.342	0.064 ± 0.095	*0.050*	−1.629 ± 0.752	**0.030**	0.477 ± 0.120	**7.91e-05**	0.062 ± 0.022	**0.005**
MCI	[−0.055; 0.039]		[−0.214; 0.075]		[−0.123; 0.251]		[−3.105; −0.153]		[0.240; 0.713]		[0.019; 0.105]
CN vs.	−0.002 ± 0.026	0.929	−0.076 ± 0.080	0.337	0.199 ± 0.103	*0.053*	−2.383 ± 0.831	**0.004**	0.943 ± 0.130	**6.75e-13**	0.106 ± 0.024	**7.20e-06**
Dementia	[−0.053; 0.049]		[−0.233; 0.080]		[−0.003; 0.401]		[−4.013; −0.753]		[0.688; 1.198]		[0.060; 0.152]

*CI, confidence interval; GM, gray matter; SD, standard deviation; TIV, total intracranial volume. The bold values indicate the significance of the CN slope and the significant difference between CN and MCI slopes as well as CN and Dementia slopes (*p* < 0.05).

### 4.2. Distinct structure-function associations in normal aging, MCI and dementia

*In the second subobjective*, we constructed regression models that predict functional performance in the tests from brain radiomics. Since structure-function association may have features specific to pathology, we trained the regression models on three study cohorts separately. As the input of the model we used the data acquired with voxel- and surface-based brain morphometry.

Examining the feasibility of employing brain morphometry for predicting neurofunctional performance in CN, MCI, and dementia cohorts, we designed an ML regression model. As the test scales differ in size, we adjusted the mean absolute error (MAE) to the range values of the tests (MAE/range, %). This allowed us to compare the accuracy of the algorithms trained on MMSE, ADAS-cog, RAVLT, TMT, and DSST. The performance of these algorithms is presented in Table 3. The test results in MMSE can be predicted much more accurately than in other tests (MAE/range = 4.5 ± 0.23 in the CN group). Despite a markedly higher mistake of the model for the ADAS-cog score (*p* = 1.84*e*−95), its prediction also had credible performance (MAE/range = 5.04 ± 0.22 in the CN group). The error of the RAVLT, TMT, and DSST score prediction was significantly higher (10.62 ± 0.5, 10.57 ± 0.68, and 10.81 ± 0.51). The dissimilarity in the accuracy of the model goes in line with the trends described in the previous subsection. In MMSE and ADAS-cog the trendlines are parallel and do not intersect. The same trends for RAVLT, TMT, and DSST merge if approximated to future life.

**Table 3:** Performance of models trained on cognitively preserved population, subjects diagnosed with MCI, or dementia with adjustment to the range of values (MAE/range, %).

**Group**	**Data**	**MMSE**	**ADAS**	**p_1 − 2_**	**RAVLT**	**TMT**	**DSST**	**p_3 − 5_**
CN	VBM	4.5 ± 0.23	5.04 ± 0.22	1.84e-95	10.62 ± 0.5	10.57 ± 0.68	10.81 ± 0.51	2.99e-142
	SBM	4.61 ± 0.23	4.96 ± 0.22	1.84e-95	10.38 ± 0.49	10.75 ± 0.67	10.24 ± 0.53	4.15e-131
	VBM+SBM	4.61 ± 0.23	4.94 ± 0.22	1.84e-95	10.07 ± 0.48	10.62 ± 0.66	10.23 ± 0.51	1.92e-129
MCI	VBM	9.28 ± 0.29	7.62 ± 0.22	3.63e-211	9.52 ± 0.3	20.13 ± 0.65	10.95 ± 0.33	1.43e-212
	SBM	9.0 ± 0.28	7.48 ± 0.21	2.46e-206	9.59 ± 0.31	18.8 ± 0.59	10.12 ± 0.33	9.38e-210
	VBM+SBM	9.06 ± 0.28	7.41 ± 0.21	1.65e-206	9.46 ± 0.3	18.81 ± 0.59	10.03 ± 0.32	1.01e-209
Dementia	VBM	13.22 ± 0.54	10.3 ± 0.42	6.98e-121	8.65 ± 0.33	26.97 ± 0.75	12.67 ± 0.46	2.14e-187
	SBM	12.67 ± 0.54	9.09 ± 0.42	3.27e-99	7.97 ± 0.34	25.75 ± 0.76	11.03 ± 0.42	3.43e-172
	VBM+SBM	12.78 ± 0.55	9.11 ± 0.41	1.75e-92	7.9 ± 0.33	25.9 ± 0.77	10.85 ± 0.42	1.94e-171

*VBM, voxel-based morphometry; SBM, surface-based morphometry.

**Figure 3:**
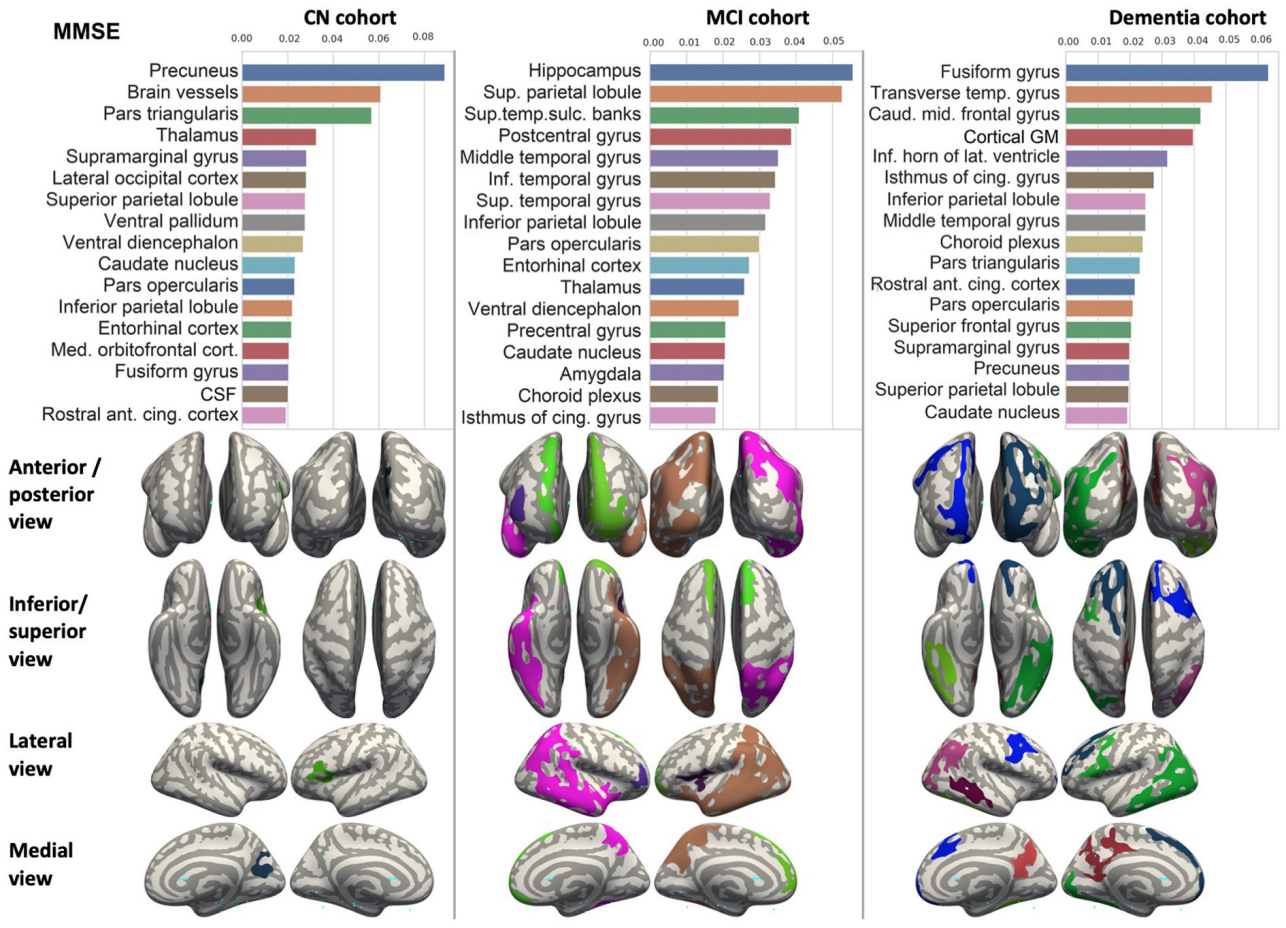
Brain structures ranked according to information gain value for MMSE score prediction. Inflated cortical representations showing significant correlations between cortical volumes and test score.

**Figure 4:**
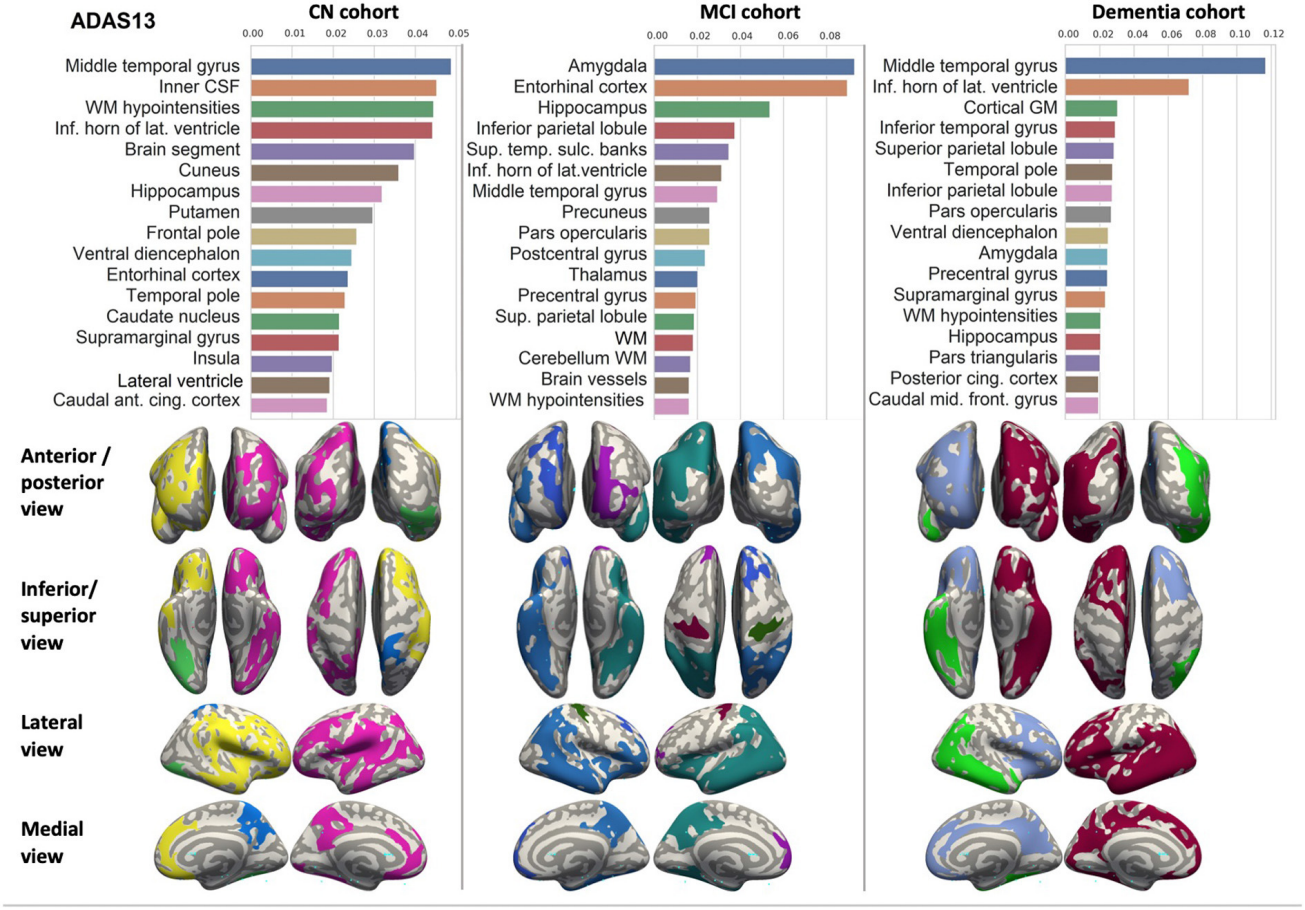
Brain structures ranked according to information gain value for ADAS13 score prediction. Inflated cortical representations showing significant correlations between cortical volumes and test score.

**Figure 5:**
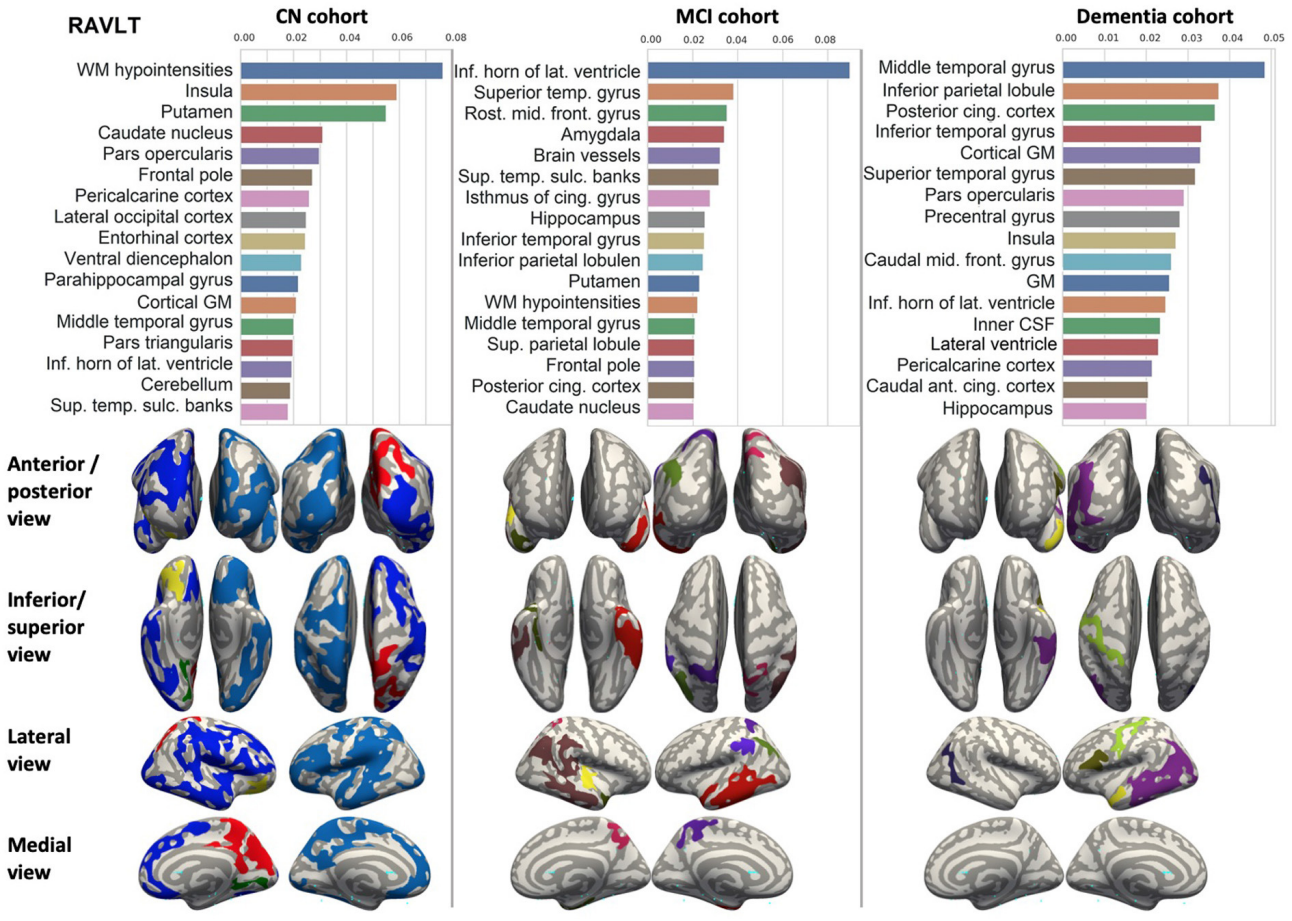
Brain structures ranked according to information gain value for RAVLT score prediction. Inflated cortical representations showing significant correlations between cortical volumes and test score.

**Figure 6:**
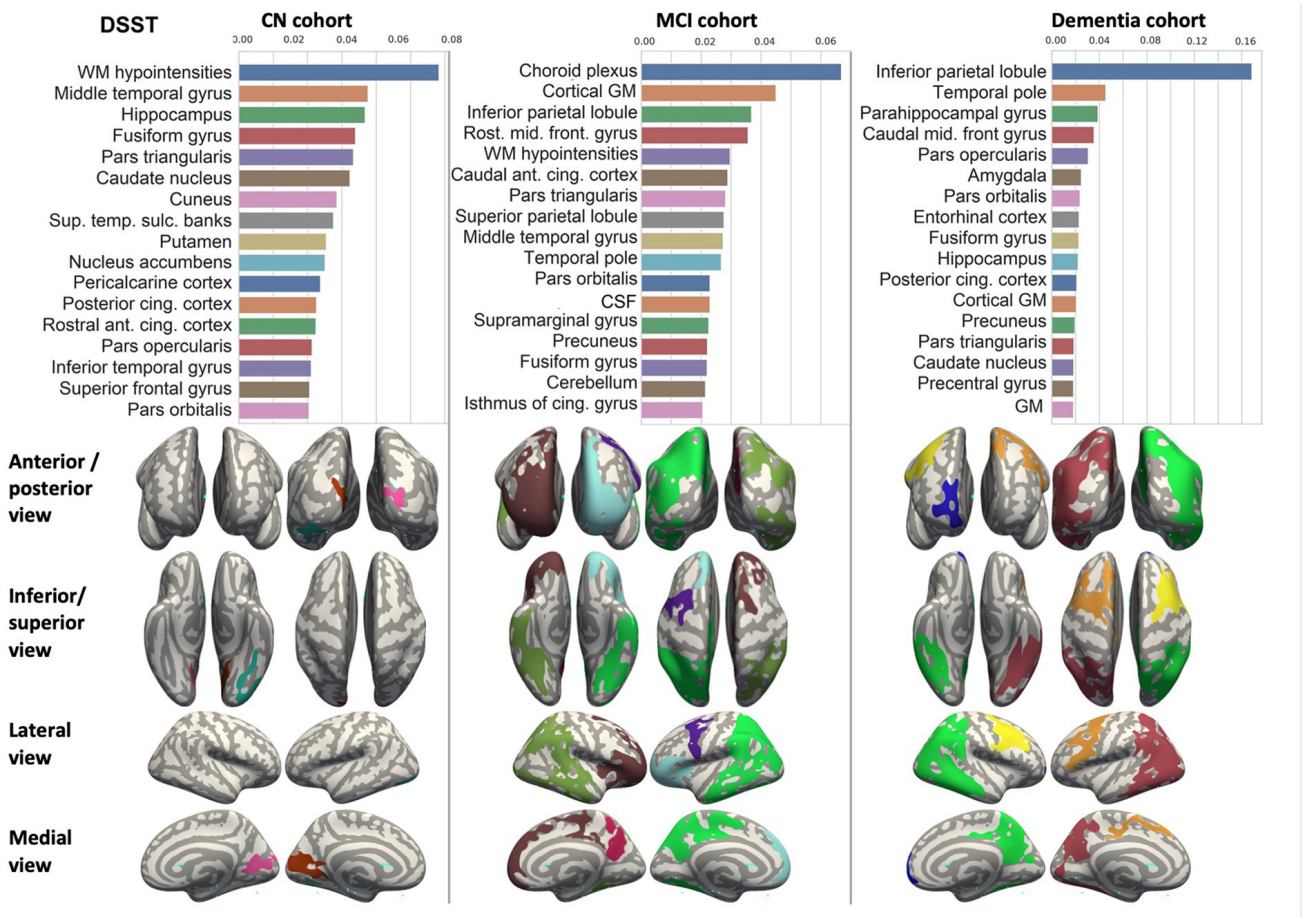
Brain structures ranked according to information gain value for DSST score prediction. Inflated cortical representations showing significant correlations between cortical volumes and test score.

**Figure 7:**
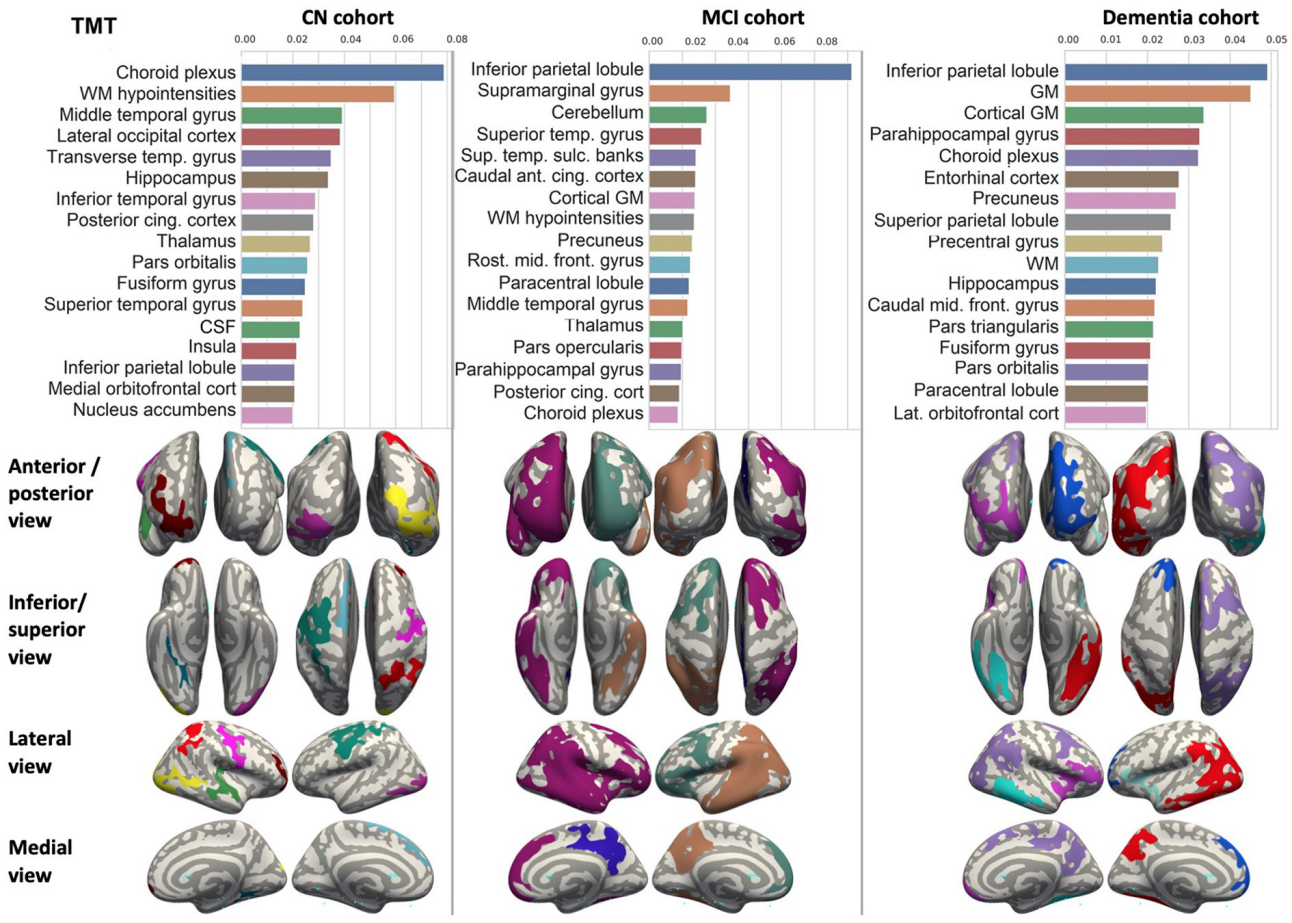
Brain structures ranked according to information gain value for TMT score prediction. Inflated cortical representations showing significant correlations between cortical volumes and test score.

We ranked the structural predictors according to the information gain value (see Figures 3–7). The column charts show that the structural determinants of cognitive performance are not equal among the tests. They also differ among the study cohorts. For example, the top valuable predictors of MMSE score are the volumes of the total brain, cerebral cortex, accumbens, cerebral white matter, inferior lateral ventricles, and hippocampus. However, the results in TMT have a weaker association with the brain structures listed above. The structural predictors of cognitive scores and results in neuropsychological tests are not identical in healthy individuals and those with cognitive impairment. In such a way, the top valuable predictors are specific to the test and to the level of decline in functioning.

In each study cohort we found clusters of cortical parcellations closely associated with performance in cognitive tests. The volume, surface area of the clusters and their number differ evidently among the studied cohorts. This is because each SBM metrics provides unique information regarding cortical anatomy and possibly different SFA patterns (Riccelli et al., 2017).

### 4.3. Classification of examinees by fitting models of brain SFA for healthy individuals, patients with MCI and dementia

**Figure 8:**
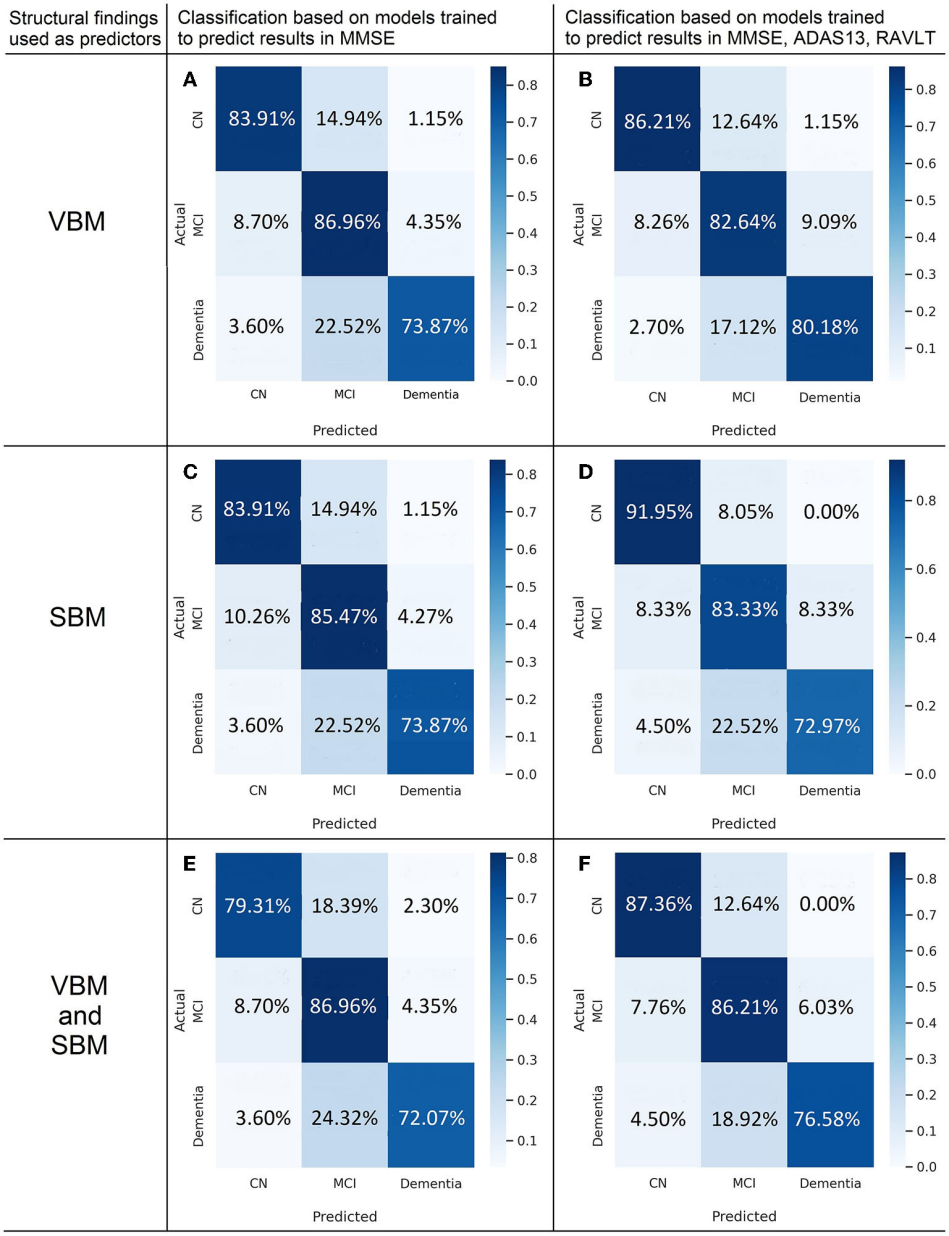
Confusion matrices of multigroup classification based on best fit to model predicting cognitive scores from voxel-based **(A,B)** and surface-based morphometric data **(C,D)** or combination of them **(E,F)**.

The highest classification accuracy is achieved with the model trained to *predict MMSE from VBM* (see Figure 8). In the cognitively normal cohort, the model identifies 85.06% of individuals as healthy subjects, and relatively small portions (14.94 and 1.15%) are misclassified as patients with MCI or dementia. The true prediction rate reaches 86.96% in the MCI group. The least accurate classification is observed in the group of the demented patients: it misclassifies over 26% of them. This is the major limitation of the constructed classification system. The diagnostic algorithm based on *ML prediction of MMSE from SBM* is almost as accurate as the previous classification (see Figure 8B). The percentage of misclassified cases in the normal cohort is slightly higher. Still, none of the cognitively preserved individuals are misclassified as demented. *When VBM and SBM predictors are used in combination*, the performance does not increase (see Figure 8C). Unexpectedly, the true predictive rate drops to 79.31 and 72.07% for the cognitively normal and demented population respectively. The inclusion of SBM predictors to the model does not boost the accuracy.

Classification based on the model trained to predict ADAS13 from VBM detects the demented patients more accurately than the other considered models at the level of 78.38% true prediction rate. The performance for the CN class is weaker in all the models predicting ADAS13.

The application of the majority voting technique to models predicting results in MMSE, ADAS, and RAVLT improved the classification performance (see Figures 8D–F). We observe the highest classification performance of the algorithm trained on SBM data for the CN group with the true positive rate of 91.95% (see Figure 8E). The accuracy of identifying MCI is 83.3%. The model trained on VBM data shows the best performance for dementia cases (true-positive rate of 80.18%, see Figure 8D). The discrimination of MCI cases from the aforementioned groups is most accurate in the model trained on both types of predictors—VBM and SBM (86.21%).

## 5. Discussion

### 5.1. Patterns of brain structure-function association indicative of MCI and dementia

Researchers studied age-related functional change in the brain and showed neuro-cognitive slowing with different tests and approaches (Statsenko et al., 2019, 2020, 2021g,h,i; Belghali et al., 2020; Gorkom et al., 2021). In this study we explored the age-related variability of cognitive scores in the tests that are most commonly used either to diagnose MCI and dementia or to improve the accuracy of multimodal diagnostics. We started with the tests of global cognitive functioning: MMSE and ADAS-cog. The distribution of the test results over age is shown in Figures 2A,B. As ADNI dataset contains follow-up studies of healthy people and patients with cognitive impairment, one can judge on the dynamics of cognitive performance by looking at the diagrams. The trends are horizontal for the performance in MMSE and ADAS-cog in all study groups. This means that the global cognitive functioning changes slightly with age in the cognitively normal population. It also remains stable across the disease course. Though there are patients with reversible or progressive MCI, the number of such cases is quite low.

The second group of tests covers a few cognitive domains, i.e., information processing in DSST, memory in RAVLT, information processing in TMT. Scores in RAVLT test are quite stable in normal aging and across the disease course with a slight trend toward lowering in all the study groups (see Figure 2C). The pace of neurocognitive slowing is moderately higher in the CN group and MCI patients. Thus, the average result for all the groups would reach a common value if the observation lasted several more decades.

The trendlines on Figures 2D,E show clear signs of malfunctioning in several cognitive domains assessed with DSST and TMT tests. The performance worsens with time. For this reason, the trendlines of CN, MCI, and AD groups converge at the approximated point of 100 years of age.

As seen from the diagrams in Figures 2D,E the trendlines of the performance in neuropsychological tests (TMT and DSST) and the degeneration of the gray matter show the same dynamics in the correspondent cohorts (see Figure 2F). But the slopes for the gray matter volume adjusted to the total intracranial volume are steeper than the trendlines for the results in DSST or TMT. Presumably, brain plasticity helps an individual to adjust to aging and disease and compensates for the loss of the gray matter volume.

### 5.2. Models of brain SFA in cognitively normal individuals, patients with MCI and dementia

SFA models projecting cognitive scores achieved different accuracy in CN, MCI, and dementia groups (see Table 4). To explain this fact, we analyzed mechanisms of developing dementia. The majority dementia cases arise from protein aggregation disorders (e.g., the accumulation of β-amyloid, τ-protein, etc.). Generic variability in the expression level of the deposited protein is important in pathogenesis of neuronal diseases. It accounts for different solubility of the aggregation-prone protein and the efficiency of clearance mechanisms that keep misfolded proteins in check. Besides, the clinical appearance of dementia varies because of selective neuronal and regional loss that differs among misfolding diseases (Fu et al., 2018).

**Table 4:** Metrics of models trained on cognitively preserved population, subjects diagnosed with MCI or dementia (MAE).

**Test**	**Data**	**CN**		**MCI**		**Dementia**		***p*-value**
		**Mean ±Std**	**CI**	**Mean ±Std**	**CI**	**Mean ±Std**	**CI**	
MMSE	VBM	0.81 ± 0.04	[0.73–0.89]	1.67 ± 0.05	[1.57–1.77]	2.38 ± 0.1	[2.19–2.57]	3.97e-239
	SBM	0.83 ± 0.04	[0.75–0.92]	1.62 ± 0.05	[1.52–1.72]	2.28 ± 0.1	[2.09–2.47]	1.34e-234
	VBM+SBM	0.83 ± 0.04	[0.75–0.91]	1.63 ± 0.05	[1.53–1.73]	2.3 ± 0.1	[2.11–2.49]	8.23e-232
ADAS-cog	VBM	3.24 ± 0.14	[2.96–3.51]	4.9 ± 0.14	[4.62–5.17]	6.63 ± 0.27	[6.09–7.16]	6.53e-239
	SBM	3.19 ± 0.14	[2.91–3.46]	4.81 ± 0.14	[4.53–5.08]	5.85 ± 0.27	[5.32–6.38]	5.12e-230
	VBM+SBM	3.18 ± 0.14	[2.9–3.45]	4.77 ± 0.14	[4.5–5.03]	5.86 ± 0.26	[5.35–6.38]	1.44e-227
RAVLT	VBM	7.33 ± 0.35	[6.65–8.01]	6.57 ± 0.21	[6.16–6.98]	5.97 ± 0.23	[5.52–6.42]	2.86e-231
	SBM	7.16 ± 0.34	[6.5–7.82]	6.62 ± 0.21	[6.2–7.04]	5.5 ± 0.23	[5.04–5.96]	4.14e-220
	VBM+SBM	6.95 ± 0.33	[6.31–7.6]	6.53 ± 0.21	[6.12–6.93]	5.45 ± 0.23	[5.0–5.9]	2.48e-213
TMT (part B)	VBM	28.32 ± 1.83	[24.73–31.9]	53.94 ± 1.73	[50.55–57.33]	72.28 ± 2.02	[68.32–76.24]	2.26e-229
	SBM	28.81 ± 1.81	[25.26–32.35]	50.38 ± 1.57	[47.29–53.46]	69.0 ± 2.03	[65.01–72.99]	5.56e-184
	VBM+SBM	28.47 ± 1.77	[25.0–31.95]	50.4 ± 1.58	[47.3–53.49]	69.41 ± 2.07	[65.36–73.47]	1.38e-180
DSST	VBM	8.43 ± 0.4	[7.65–9.21]	8.54 ± 0.26	[8.03–9.04]	9.88 ± 0.36	[9.18–10.57]	4.88e-230
	SBM	7.99 ± 0.41	[7.19–8.79]	7.89 ± 0.25	[7.4–8.39]	8.6 ± 0.33	[7.95–9.24]	3.28e-192
	VBM+SBM	7.98 ± 0.4	[7.19–8.77]	7.82 ± 0.25	[7.32–8.31]	8.46 ± 0.33	[7.81–9.1]	6.89e-187

*VBM, voxel-based morphometry; SBM, surface-based morphometry.

In all the tests, the informative value of brain structures in the prediction of cognitive scores differs by the study group (CN, MCI, dementia). This justifies that the healthy cohort and patients with a pathology have specific SFA patterns. We analyzed the patterns in the demented patients of ADNI dataset and discussed the findings. As AD accounts for the majority of dementia cases, we found the structures vulnerable for change in β-amyloidopathy. Other neurodegenerative diseases selectively damage different groups of neuronal cells and brain regions, which would result in other SFA patterns. To explain the structural determinants of cognitive performance, we analyzed the involvement of definite brain areas in the tests (see Tables 5, 6). The structural predictors of cognitive scores are described in details in Supplementary material.

**Table 5:** Structural brain correlates and cognitive functions involved in MMSE, ADAS-cog, and RAVLT.

**Test**	**Cognitive functions**	**Relevant brain structures**
**MMSE**
	Temporo-spatial orientation
		Precuneus
		Cortical gray matter
	Memory recall
		Precuneus
		Intracranial arteries
		Hippocampus
		Cortical gray matter
	Concentration
		Superior parietal lobule
		Cortical gray matter
	Language
		Pars triangularis
		Hippocampus
		Caudal middle frontal gyrus
		Cortical gray matter
	Visuospatial function
		Precuneus
		Superior parietal lobule
		Fusiform gyrus
		Caudal middle frontal gyrus
		Cortical gray matter
	Working memory	Precuneus Intracranial arteries Hippocampus Cortical gray matter
**ADAS-cog**
	Temporo-spatial orientation	White matter lesions
	Memory and new learning: - Word recall - Orientation - Word recognition - Memorizing test instructions	Mesial temporal lobe White matter lesions Inferior lateral ventricles Hippocampus Putamen Amygdala Entorhinal cortex
	Language: - Commands - Spoken language ability - Naming objects / fingers - Word-finding difficulty - Comprehension	White matter lesions Inferior lateral ventricles Putamen Hippocampus
	Praxis: - Constructional praxis - Ideational praxis	White matter lesions
	**RAVLT**	Episodic memory	White matter lesions Insula Inferior lateral ventricles Mesial temporal lobe Inferior parietal lobe Posterior cingulate cortex
		Attention	Putamen Inferior lateral ventricles Mesial temporal lobe Inferior parietal lobe Posterior cingulate cortex

**Table 6:** Structural brain correlates and cognitive functions involved in DSST and TMT.

**Test**	**Cognitive functions**	**Relevant brain structures**
**DSST**	Motor speed	White matter lesions Caudate nucleus Cortical gray matter Inferior parietal cortex
		Working memory	White matter lesions Mesial temporal lobe Hippocampus Cortical gray matter Inferior parietal cortex Rostral middle frontal gyrus
		Attention	White matter lesions Mesial temporal lobe Hippocampus Caudate nucleus Cortical gray matter Inferior parietal cortex Rostral middle frontal gyrus
		Associative learning	White matter lesions Mesial temporal lobe Hippocampus Cortical gray matter
		Visuoperceptual abilities: - Scanning - Capacity to write/draw.	White matter lesions Mesial temporal lobe Hippocampus Fusiform gyrus Cortical gray matter Inferior parietal cortex
	**TMT**	Visuoperceptual abilities: - Visual scanning - Visual-conceptual tracking - Visual-motor tracking	White matter lesions Mesial temporal lobe Inferior parietal cortex
			Information processing	White matter lesions Superior marginal cortex
			Attention	White matter lesions Mesial temporal lobe Inferior parietal cortex
			Motor speed	White matter lesions Inferior parietal cortex
			Memory: - Working memory - Rote memory	White matter lesions Mesial temporal lobe Inferior parietal cortex

#### 5.2.1. MMSE, ADAS-cog, RAVLT

The prediction of neuropsychological profiles from radiologic and nuclear medicine findings was the issue of recent studies by other authors. They used different imaging modalities, e.g., resting-state functional MRI (Duc et al., 2020), structural MRI (Kovacevic et al., 2009; Stonnington et al., 2010; Yan et al., 2015; Moradi et al., 2017; Beyer et al., 2019; Imani et al., 2021), and PET (Zhang et al., 2012; Beyer et al., 2019). In those studies, neuroscientists trained regression models to compute *MMSE*, *ADAS*−*cog*, and *RAVLT*_*immediate*_ scores at the time of examination (Duc et al., 2020), in 6 months (Kovacevic et al., 2009), 12 months (Imani et al., 2021), 24 and 36 months after it (Zhang et al., 2012). The authors also built SFA models but for different purposes, i.e., they aimed to forecast the disease course, to calculate the *MMSE* score and diagnose AD disease status from MRI. They wanted to get the *MMSE* score without trained clinicians. In contrast, we proposed to combine the findings collected by radiologists and qualified neurologists for the advanced early diagnostics of MCI or AD. We did not aim to forecast the disease progression.

In our study, the most accurate model (the one with the minimal MAE/max.score) predicts *MMSE* score. This model has reputable performance: the minimal MAE in AD population (2.3 ± 0.1) is lower than in the study by Duc et al. (2020). The minimal MAE of the *RAVLT*_*immidiate*_ models in MCI is 6.53 ± 0.21, in AD—5.45 ± 0.23. This is lower than in the study by Moradi et al.: 6.92 ± 0.035 and 5.75 ± 0.07, respectively (Moradi et al., 2017). The authors did not built the models for healthy adults. In our study, such models have optimal performance and help us to distinguish individuals without the disease from MCI and AD cohorts. The computations of *ADAS*−13 values in our study has the minimal MAE of 3.18 ± 0.14 in CN, 4.77 ± 0.14 in MCI and 5.45 ± 0.23 in dementia. The accuracy is close to the mean performance metrics of the models trained by V. Imani to predict the score changes in the same scale for cognitive assessment: 3.07, 3.87, and 5.01 (Imani et al., 2021).

#### 5.2.2. DSST and TMT tests

Researchers studied neuroanatomical predictors of results in DSST (Bruno et al., 2016; Lazari et al., 2021) and TMT tests (Van De Pol et al., 2007; Oosterman et al., 2010). These studies were focused on the associations of MRI measures with cognitive performance and confirmed a tight association between medial temporal lobe atrophy and DSST, ADAS-cog scores (Van De Pol et al., 2007) as well as TMT-B performance (Oosterman et al., 2010). They also justified the hippocampal integrity which was derived from the ratio of parenchyma volume over total volume as an informative predictor of future changes in general cognitive ability. Those changes were assessed with composite cognitive score calculated from MMSE, DSST, and RAVLT delayed recall (Bruno et al., 2016). The authors of the aforementioned studies highlighted the importance of structural covariance in the prediction of individual differences in executive function skills. However, they did not report the performance of a regression model which would accurately forecast the test from MRI or other structural findings. Thus, our paper adds to the growing literature on the neural correlates of cognition in adults and identifies neuroanatomic coupling as a biological substrate that may contribute to executive function and dysfunction in MCI and AD.

### 5.3. Classification of examinees into cohorts according to the SFA pattern

The multimodal approach is supposed to be more accurate than the unimodal one. The combined analysis of medical findings with individual risks advance the diagnostics and support the personalized therapy. The recent findings by other authors as well as our results convincingly show this (Habuza et al., 2021a; Statsenko et al., 2021a,b,c, 2022; Al Zahmi et al., 2022).

### 5.3.1. Accuracy of unimodal approach

#### 5.3.1.1. Cognitive tests

Due to the limited availability of other diagnostic methods, cognitive tests continue to be the most commonly used *screening* for neurodegenerative diseases. In the optimal therapeutic tactics, clinical psychologists test patients for MCI and dementia. Cognitive tests are time consuming, their reliability is disputable. According to the previous studies, the accuracy of the tests distinguishing MCI from healthy aging ranges from 58 to 90% (Tóth et al., 2018; Müller et al., 2019; Fernández-Fleites et al., 2021; Rashedi et al., 2021). Because of the inconsistent findings clinicians cannot rely on cognitive scores as a single diagnostic modality (Statsenko et al., 2021e,f). Physicians order *magnetic resonance imaging (MRI) examination* to evidence the diagnosis and to perform differential diagnostics.

#### 5.3.1.2. Neuropsychological tests

The optimal diagnostic modality should be reliable, economically affordable, operator-independent, easy, and quick to perform. Neuropsychological tests partially meet the criteria mentioned above: they are not expensive, easy to administer, quick to pass, and to assess the results. They can aid in differentiating MCI from AD and identifying cognitive deficits related to preclinical AD and MCI (Collie and Maruff, 2000).

The tests differ significantly in the accuracy of classification of MCI patients and cognitively intact adults. The sensitivity of dementia diagnostics with MMSE ranges from 44 to over 80% (Kalbe et al., 2004; Shankle et al., 2005; de Jager et al., 2009). Studies which compared MMSE with other cognitive tests led to conflicting findings. In a study, the sensitivity of MCI detection with the Persian version of MoCA was 86 and 72% with MMSE (Rashedi et al., 2021). Another study reported MMSE to be the most sensitive instrument followed by the delay recall test, and the Montreal Cognitive Assessment (Hemmy et al., 2020). The traditional cut score of 26 in MoCA showed the best classification accuracy (Rossetti et al., 2019). A delayed recall in the verbal fluency and episodic memory tests were shown to be reliable predictors of AD (Arnáiz and Almkvist, 2003).

#### 5.3.1.3. MRI

Although there are structural similarities in normal aging and MCI patients, MRI remains a method of choice in diagnosing MCI (Taheri Gorji and Kaabouch, 2019; Statsenko et al., 2021d; Uzianbaeva et al., 2021). Structural MRI detected change in the brain in MCI patients at early stage with 78.8% Sn and 77.1% Sp. The performance of classification algorithms was boosted by nearly 20% when structural MRI was combined with mean diffusivity and fractional anisotropy MRI (Kang et al., 2020). Since volumetric change in the hippocampus is a marker of AD, it can also serve as a sign of MCI. Models trained on volumetric measures of the hippocampus distinguished between MCI and healthy groups with 69% Sn and 73% Sp. When trained on combined volumetric data for the hippocampus and other brain regions, the model had a lower Sn—66% (Westman et al., 2011). Abnormal thinning of the cortex is another marker of MCI or AD. A study showed that 17 structures could be used to classify *early MCI* patients and healthy controls. With the *MCI progression*, the number of informative brain regions increased to 22. These features could classify various stages of MCI and healthy controls with nearly 75% Sn and Sp depending on algorithms (Rallabandi et al., 2020).

Reporting and classifying cases with regard to the pathology is a challenging task for radiologists and for computer vision systems. For instance, the accuracy of identifying healthy examinees with structural MRI modalities was around 70–73% (Westman et al., 2012; Kang et al., 2020). It was roughly similar to the accuracy of detecting MCI progression to AD with the same type of data (Westman et al., 2012; Willette et al., 2014). Some authors reported the accuracy of the automatic segregation between stable MCI and AD at the level of 85–86% (Basaia et al., 2019). However, the number of reports with such optimistic data is low and the reliable computer aided diagnostic system is not yet available in the real clinical settings. In another study, MRI differentiated between MCI and AD patients with Sn and Sp of above 80% (Basaia et al., 2019). Recent studies suggested that temporal lobe changes at early stage of AD and volumetric measurements of the region could help in distinguishing between MCI and AD. The models trained on MRI images of the amygdala and hippocampus discriminated AD from MCI with 87.2% Sn (Bottino et al., 2002). The performance of the models trained exceptionally on the hippocampal images was lower (Westman et al., 2011).

#### 5.3.1.4. Nuclear medicine

Many researchers struggle to find sensitive markers that would allow them to detect mild cognitive impairment (MCI) at early stages or to identify the progression of MCI to AD. Some authors report promising findings on the success in advanced imaging modalities [positron emission tomography (PET), single-photon emission computerized tomography (SPECT)] and molecular markers [β-amyloid in cerebrospinal fluid (CSF)]. For instance, the accuracy of early diagnostics of AD with SPECT ranged from 70 to 90% in different references (Seto et al., 2021; Wang et al., 2021). The same method can also be used for the differential diagnostics between AD and the frontotemporal dementia with the accuracy of about 84% (Horn et al., 2009) and from vascular dementia with the accuracy up to 75% (Dougall et al., 2004). Various radiotracers used in the studies can account for disparity in the results. Despite the high reliability of nuclear medicine findings, the applicability of these study methods in the real clinical settings is low. No fund covers a broad population screening for cognitive impairment with such methods.

#### 5.3.1.5. Electrophysiology

EEG can detect pathologic change in the brain electrical activity caused by cognitive impairment. It detects abnormalities in synchronization/desynchronization and coupling/decoupling of neural activities in AD patients. Quantitative EEG is a useful tool for differentiating between AD, vascular dementia and healthy aging. The tool can predict cognitive decline in healthy people with 90% accuracy (Prichep et al., 2006). Recent studies showed a possibility to stratify patients with Alzheimer's dementia and MCI with event-related EEG. Event-related potentials predict the progression of MCI to AD with 70–78% accuracy (Babiloni et al., 2020).

### 5.3.2. Multimodal diagnostics and screening

Some researchers reported a slight improvement in classification performance after combining neuropsychological test scores with MRI findings. The diagnostic model trained on ADAS1, visual delayed recall and left hippocampal measures had 84% Sn and 81% Sp (Liu et al., 2011). In another study, models trained on MRI had 59.6% Acc, on the results in neuropsychological tests—89.8% and on a combination of MRI findings and test scores—90.1% (Goryawala et al., 2015). These findings stay in line with the hypothesis and final results of the current study.

In contrast to our study, many researchers use the same multimodal approach for other purposes: they forecast the disease course from various types of diagnostic data. For example, they build multimodal diagnostic models for predicting MCI-to-AD conversion. A report showed an insufficient accuracy of the classification based on neuropsychological and SPECT data (33% Acc, 100% Sn, 33% Sp; Quaranta et al., 2018). In another study, a joint analysis of memory scores and SPECT images had the Sn and Sp of 77.8% (Borroni et al., 2006). A research team created a multimodal system that incorporated the cognitive scores and ApoE genotypes (62% Acc) with MRI, PET, and CSF data (81% Acc) (Yu et al., 2012). A similar study used the MRI and PET images, CSF biomarkers, and gene data as the input data. It reported the classification accuracy of 84.7% (Lin et al., 2020). The models that forecasted the MCI progression from a combination of PET findings with cognitive scores exhibited a considerable improvement in accuracy (up to 95.65%) (Teng et al., 2020). Some researchers studied the additive value of distinct diagnostic modalities. They showed the classification accuracy of models trained on MRI data (63.9% Acc, Sn 76.7%, Sp 54.8%). The models had an improved performance when supplied with PET and genetic data (68.15, 83.3, and 57.1%, respectively). The accuracy raised even more when the invasive CSF study was added (68.1% Acc, 90.0% Sn, 52.4% Sp) (Young et al., 2013). While SFA patter is more commonly used to forecast the disease outcomes, in our study it was applied for diagnostic purposes. This was a distinguishing feature of our approach and we showed its potential.

## 6. Strength and limitations

The major *limitation* of the study is that the authors analyzed only cases of Alzheimer's MCI or dementia. The proposed approach can be used for screening rather than diagnostic purposes. Future research is required to adopt the classification model to other clinical forms of dementia. The *strength* is as follows. For the study, we used findings of non-invasive cognitive tests and MRI examination, in particular, a routine structural MRI (a 3D T1-weighted scanning sequence). The accessibility of the equipment required for such a study is high. Therefore, the results of the study can be easily applied into practice.

## 7. Conclusion

- In healthy aging, the global cognitive functioning changes slightly. It also remains stable across the course of neurodegenerative diseases with the exception of uncommon reversible or progressive cases. Scores in RAVLT are quite stable in normal aging and across the disease course with a minor downward trend in all the study groups. The pace of neurocognitive slowing is moderately higher in the CN group and MCI patients. The difference in pace of changes results in a converging trend. Thus, if the observation lasted several more decades, the average result for all the groups would reach a common value. Within time, there appear clear signs of worsening the performance in several cognitive domains assessed with DSST and TMT. The trendlines of the CN, MCI, and AD groups converge at the approximated point of 100 years of age.
- We constructed regression models that predict functional performance in cognitive tests from brain radiomics. In accelerated aging, the neuronal loss differs among distinct cell groups and brain regions. Logically, the SFA may have features specific to the pathology. The models that we built reflect specific SFA patterns for each study cohort. We used the feature selection technique to identify the most informative structural neuroimaging measurements.
- According to the SFA pattern, we distinguish three cohorts: the cognitively normal elderly, patients with MCI and Alzheimer's dementia. The highest accuracy is achieved with the model trained to predict MMSE from voxel-based morphometry data. In the cognitively normal cohort, the model identifies 85.06% of individuals as healthy subjects, and relatively small number of cases (14.94 and 1.15%) stays misclassified. In the MCI group, the true prediction rate reaches 86.96%. The demented patients are identified less accurately (73% Acc), which is the major limitation of this approach. The classification based on the model trained to predict ADAS13 from VBM detects the demented patients more accurately than other models (78.38% true prediction rate).
- The majority voting technique applied to models that predict results in MMSE, ADAS, and RAVLT improved the classification performance. In the CN group, we observed the highest classification performance of the algorithm trained on SBM data with the true positive rate of 91.95%. The discrimination of MCI cases is most accurate in the model trained on both types of predictors—VBM and SBM (86.21%). The model trained on VBM data shows the best performance for dementia cases (true-positive rate of 80.18%). Thus, the multimodal approach described in this study may advance the screening for MCI and Alzheimer's dementia.

## 8. Code availability statement

The code developed for this study is available on request at: bi-dac.com.

## Data availability statement

Publicly available datasets were analyzed in this study. The data used in this study were obtained from the Alzheimer's Disease Neuroimaging Initiative (ADNI) database.

## Ethics statement

Ethical review and approval was not required for the study on human participants in accordance with the local legislation and institutional requirements. Written informed consent for participation was not required for this study in accordance with the national legislation and the institutional requirements.

## Author contributions

YS and TH contributed to the conceptual idea of the paper. YS formulated the objectives. YS and SM wrote the manuscript. TA, KG, JG, and ML contributed to the literature review and data analysis. TH performed the statistical analysis, prepared the figures and tables for data presentation, and illustration. All authors contributed to the article and approved the submitted version.

## Footnotes

^1^Data used in preparation of this article were obtained from ADNI database (adni.loni.usc.edu). As such, the investigators within the ADNI contributed to the design and implementation of ADNI and/or provided data but did not participate in analysis or writing of this report. A complete listing of ADNI investigators can be found at: http://adni.loni.usc.edu/wp-content/uploads/how_to_apply/ADNI_Acknowledgement_List.pdf.
