# Supplementary material for: Patterns of structure-function association in normal aging and in Alzheimer's disease: Screening for mild cognitive impairment and dementia with ML regression and classification models
Source: Front Aging Neurosci. 2023 Feb 23;14:943566. doi: 10.3389/fnagi.2022.943566 (PMC9995946; doi:10.3389/fnagi.2022.943566)
Supplement: Supplementary file 1 [file Data_Sheet_1.pdf]

Supplementary Material

5.2.1 Structural predictors of MMSE score

The test is one of the most frequent tools for screening cognitive impairment in older adults. It is also used to evaluate cognitive impairment progression in follow-up visits. MMSE examines various cognitive domains: temporo-spatial orientation, memory recall, concentration, language, visuospatial function, and working memory. The top valuable structural predictors of MMSE results are listed in Figure 3 and described below.

Precuneus volume is the most informative predictor. It is a cortical region located in the posterior portion of the medial parietal cortex. Recent functional imaging findings in healthy subjects suggest its involvement in a wide spectrum of highly complex functions, including visuo-spatial imagery, episodic memory retrieval, working memory, and orientation (1, 2). Consequently, its integrity determines successful achievement of several MMSE tasks, such as the intersecting pentagon copying test, short-term memory recall, and orientation to time and place.

The relative volume of intracranial arteries is the second in the list of structural parameters with greatest prognostic gain. The lumen of the vessels decreases in atherosclerosis which is associated with impaired cognitive function due to reduced cerebral blood flow and ischemic damage. A recent study reported that intracranial stenosis of arteries and increased plaque number correlate with more lacunes, larger volume of white matter lesions and memory decline (3, 4). Hence, the decreased total volume of intracranial arteries is implicated in the performance deficiency in the word registration-repetition task and short-term memory recall of MMSE test.

The pars triangularis relative volume is the third index that produces the most informative input. The structure refers to the triangular shaped cortical region of the inferior frontal gyrus in the frontal lobe. It is a segment of Broca's area which takes part in expressive aspects of the spoken and written language. More characteristically, the pars triangularis is involved in the semantic processing of language and syntax. The relatively high predictive value of the pars triangularis in MMSE can be explained by its essential participation in the language tasks: sentence repetition, instructions comprehension, reading sentences and doing as they say, writing short sentences, recognition and naming of two common objects.

MMSE score in patients with MCI. In the MCI cohort, *the hippocampus* contributes the most to the information gain. Its involvement in working memory, memory recall and language processing and production might affect the successful fulfillment of the following tasks: repetition, instruction comprehension, recognition and naming of objects, spelling "WORLD" backward, and short term memory recall. *The superior parietal lobule* is the next best estimator for MMSE test scores. It is topographically close to the occipital lobe and is employed in some aspects of concentration and visuospatial perception. Its participation in the tasks of copying pentagons and spelling "WORLD" backward might explain the high informative value.

MMSE score in patients with dementia. MMSE test scores in the dementia patients are best forecasted from the volumes of the brain parts different from those observed in the CN and MCI cohorts. *The fusuiform gyrus* has the greatest informative potential in the dementia group. Its function mainly comprises visual processing, i.e., word and object recognition, visuospatial perception. Hence, it's a key partaker in the tasks of copying of intersecting pentagons, reading a sentence, recognising and naming common objects. Next, the transverse temporal gyrus, caudal middle frontal cortex, and cortical grey matter have the highest information gain. *The transverse temporal gyrus* is the first cortical region to process incoming auditory information. Thus, its impairment could be associated with less efficient processing of speech-related

stimuli, which could, in turn, impede learning and perceiving speech sounds thus affecting the performance in sentence repetition and instruction comprehension tasks of MMSE. *The caudal portion of the middle frontal gyrus* contains the frontal eye fields which control saccadic eye movements. It is these movements which make it possible to scan numerous details within a scene. The role of the structure in visual attention might be relevant to the visuospatial and language tasks including recognition of common objects, reading a sentence and doing as it says, copying intersecting pentagons. Finally, *the grey matter of the cerebral cortex* also has a relatively high information gain value. Since it comprises the four lobes, i.e., frontal, parietal, temporal, and occipital, it is involved in a wide range of cognitive processes. The cortical grey matter (GM) participates in memory and learning, sensory perception such as seeing, hearing, speech, language comprehension, concentration, visuospatial processing, orientation, spatial attention and mapping. Hence, its integrity is required for the successful completion of all MMSE tasks.

5.2.2 Structural predictors of ADAS13 score

ADAS is a brief cognitive test battery that evaluates learning and memory, language production, language comprehension, constructional and ideational praxis, etc. In this study we resorted to average values of ADAS - ADAS13. The reason for the choice of this dependent variable is explained in Subsection 4.1. The information gain value of the brain volumes in predicting ADAS13 in CN, MCI and dementia cohorts is illustrated in Figure 4. For CN subjects, *the middle temporal volume* yields the highest information gain value. *The hippocampus, amygdala* and other structures constitute the medial temporal lobe which is essential for episodic memory. Encoding, consolidation, and retrieval are the processes composing the memory function of the lobe while word recall and remembering test instructions are among the tasks assessing memory in the ADAS-cog test.

Intraventricular CSF is the second top-informative predictor of performance in ADAS-cog in the CN cohort. The rise in the volume suggests larger ventricles and indicates brain atrophy which hampers cognitive abilities. Larger ventricles are strongly correlated with lower white matter integrity due to the small vessel disease (5). Increasing intraventricular CSF volume is significantly associated with increasing severity of cognitive impairment and reaction time in the tests.

White matter (WM) hypointensities and the inner CSF are predictors of roughly the same informative value. The hypointensities are areas of attenuated signal on T1-weighted MRI scans. Pathological lesions in these regions include myelin pallor, tissue rarefaction associated with a loss of myelin and axons, and mild gliosis (6). They are also associated with a faster deterioration in global cognitive performance as well as in memory, learning, praxis, and language (7). These functions are believed to depend on some brain structures including subcortical neural networks and cortical-subcortical circuits. The latter can be damaged while passing through the WM. Reasonably, the WM integrity is the key index to predict ADAS13.

Inferior horns of the lateral ventricles have the information gain value almost identical to that of the inner CSF and WM hypointensities. Inferior lateral ventriculomegaly is caused by passive enlargement of the inferior (temporal) horns of the lateral ventricles. Typically, the enlargement follows neuronal loss and brain parenchymal atrophy in the temporal lobe. Therefore the temporal horns have been repeatedly used as the index for middle temporal lobe atrophy (8) which is the top informative biomarker in our study. They may also reflect the level of cognitive impairment. Accordingly, subjects who suffer from cognitive decline exhibit greater temporal horn enlargement compared to their cognitively stable counterparts. Notably, the information gain of the inferior horns is twice as high as that of the total lateral ventricles. This can be explained by the fact that the temporal horn volume is a measurement of the middle temporal lobe atrophy while the size of lateral ventricles is indicative of the global cerebral atrophy. Consequently, it is less

specific for neuropsychological decline compared to the temporal horns and has lower significance in projecting ADAS.

The fifth most informative predictor is *the ratio of the brain segment to the total intracranial volume*. The brain segment includes voxels of all intracranial structures with the exception of the brain stem and background. It also includes the vessels, optic chiasm and CSF.

The information gain of the *cuneus* is approximately 75% bigger than that of the lateral ventricles. The cuneus is a wedge-shaped area on the medial surface of the occipital lobe. It is most known for its role in primary visual processing (receipt, segmentation, and integration of visual input) and secondary visual processing (analysis and discrimination of visual information in terms of motion, shape, and position). These functions are necessary for the accurate and prompt completion of ADAS tasks such as naming and word recognition. The cuneus is also involved in reward response, anticipation, attention, and working memory manipulations. Thus, it contributes to higher cognitive functions involving visual information.

The information value of *the hippocampus* is two times lower than that of the total middle temporal lobe. The hippocampus is a complex brain region crucial to semantic, episodic, and spatial memory, learning, and language comprehension and production. A decrease in hippocampal volumes is strongly associated with worse ADAS scores. To accomplish pattern recognition and memory encoding tasks of ADAS-cog, the hippocampus acquires an input from the entorhinal cortex. It is better to combine the volumetric analysis of these substructures that comprise the middle temporal lobe than to explore each of them individually.

The next information valuable structure is *the putamen* - a deep brain nucleus and a component of the basal ganglia. Through the cortico-striato-thalamocortical neural pathways, the putamen is involved in language learning functions and motor execution, including speech articulation. Its impairment leads to hindered fluency, dysarthria with clumsy hands and other clinical manifestations. ADAS requires intact motor responses, language and cognition to successfully fulfil different tasks including constructional praxis and spoken language ability. Therefore, the putamen is related to the ADAS-cog score.

The volumes of *the frontal lobe, ventral diencephalon, entorhinal and temporal lobe* also provide information for the prediction of ADAS13, since they contain neuro-centres for language, voluntary movement, object and language recognition. Although the parahippocampal gyrus correlates significantly with language and praxis subscale scores, it has a relatively low information gain value in our models predicting ADAS13.

ADAS13 score in patients with MCI. The middle temporal lobe volume is not high informative in the MCI group. Instead, each of its three main components - the amygdala, the entorhinal cortex and the hippocampus - achieves the highest values of the parameter. The middle temporal lobe is composed of several structures that can disproportionately contribute to the projection of ADAS. In our study, *the amygdala* is the strongest predictor of ADAS13 in the MCI group. It is known for its key role in regulating emotions and encoding memory of them. The predictive value of the amygdala volume can be justified by the fact that emotions impact several cognitive processes, including memory and learning. *The entorhinal cortex* has the second greatest predictive gain in the performance of the model. Working memory, spatial learning and memory are among the functions of the structure. Injury to the entorhinal cortex can impact efficiency in such ADAS-cog tasks as word recall, remembering test instructions and orientation. *The hippocampus* ranks third among the best estimators in the MCI cohort. Its contribution to learning; language comprehension and production; semantic, working and spatial memory makes it crucial for fulfilling multiple ADAS tasks.

ADAS13 score in patients with dementia. In demented patients, the volume of the middle temporal lobe and the inferior lateral ventricles are the top predictors of ADAS13 in our models.

5.2.3 Structural predictors of RAVLT score

RAVLT is a powerful neuropsychological instrument for assessing episodic memory and attention. It evaluates the ability to learn 15 words in five immediate trials, to remember the words after an intervening interference list, then to recall and recognize the words after a 30-minute latency interval. RAVLT is commonly used to test cognitive abilities in dementia and pre-dementia patients. Figure 5 exhibits the rank of different brain regions in predicting RAVLT values.

In the cognitively preserved subjects, the best predictor is WM hypointensities. According to previous neuroimaging studies, white matter damage increases with aging and cerebrovascular disease, and is linked to episodic memory impairment in cognitively normal older individuals (9, 10). Age-related episodic memory deficits are caused by network disruption because injury to various pathways leads to the disconnection between the frontal and temporal cortex and frontal-subcortical WM tracts.

Next, the volume of *the insula* is the second best estimator of the RAVLT score. The insular cortex is a slender band of GM that is located just beneath the lateral brain surface, connecting the temporal lobe to the inferior parietal cortex. The structure is linked to verbal episodic memory tasks, which justifies our findings.

The putamen is the ensuing strongest predictor in CN cohorts. Although our understanding of the putamen's role in cognitive functioning is still limited, recent discoveries suggest that its damage results in poorer attention (11). The assumed reason for this is its involvement in the cortico-striatal-thalamo-cortical pathway that consists of connections between the basal ganglia, thalamus and multiple brain regions involved in cognitive control in the prefrontal, parietal and temporal lobes. Therefore, alterations to this region could be accountable for the impeded execution of the three tasks in RAVLT neurofunctional test.

RAVLT score in patients with MCI. *The inferior lateral ventricles* exceed all analyzed brain structures in estimating RAVLT scores in the MCI cohort. Since they reflect the temporal lobe volume, the ventricles can be used as indicators of episodic memory and attention.

RAVLT score in patients with dementia. *The middle temporal lobe* outperforms all other brain areas in calculating RAVLT scores. *The inferior parietal lobe* and its part - *the posterior cingulate cortex* - take second place in the list of predictors. The involvement of the aforementioned structures in episodic memory and attention helps us to explain the findings.

5.2.4 Structural predictors of DSST score

This is a psychomotor test that requires the participant to match symbols to numbers according to the key at the top of the page. The DSST is short and valid, that is why it is widely used in neuropsychology. It assesses a variety of cognitive functions. Motor speed, working memory, attention, associative learning, and visuoperceptual functions, such as scanning and the capacity to write or draw, are required for good DSST performance.

The structural parameter with the most pronounced information gain is *white matter hypointensities* (see Figure 6). Lower scores in the pencil and paper DSST are substantially correlated with a greater volume of WM lesions (12). A study showed a notable interaction between WM lesion and accuracy, working memory, associative learning, psychomotor speed, attention, and visuospatial functioning (12, 13). *The middle temporal lobe* and *hippocampus* have the next highest ranking. They play a crucial role in learning coordination, working memory, attention, and spatial perception which are essential for satisfactory performance in DSST test (14).

The next informative predictor is *the fusiform gyrus* - a vast structure in the inferior temporal cortex. Its role is higher-level processing of visual information, including identification and differentiation of objects, word recognition, and perception. Therefore, the successful completion of the DSST partially depends on the fusiform gyrus integrity.

The pars triangularis receives a rank equal to that of the middle temporal lobe and hippocampus. It is challenging to justify this result since the pars triangularis is involved in the language functions irrelevant to the DSST. *The caudate nucleus* ranks lower than the pars triangularis. This is supported by multiple studies that revealed correlations between the decreased volumes of the caudate nucleus, reduced attention and motor speed (15).

DSST score in patients with MCI. In the MCI cohort, *the choroid plexus* is the metric that provides most information for anticipating DSST scores. It is involved in producing the CSF and certain proteins as well as transporting solutes to the brain. The choroid plexus volume has been reported to increase with advancing age. The supposed reasons for this are, first, modifications in the choroid plexus microstructure or function and, second, ventriculomegaly (16). These lead to dysfunction in CSF synthesis and clearance as well as reduce the levels of anti-inflammatory proteins. The disruption of the neuroimmune axis eventually hampers brain homeostasis and leads to cognitive deterioration (17, 18). Due to scarcity of research in this topic, it is challenging to pinpoint which cognitive domains are impaired with structural alterations to the choroid plexus. Therefore, it is difficult to identify the DSST tasks that are affected by change in the volume of the structure.

The brain structures that receive the next rank are *the cortical GM* and *inferior parietal lobe*. The cortical GM is critical for all the cognitive domains assessed by DSST. The inferior parietal cortex plays a crucial role in auditory-spatial working memory, motor speed, attention, and visuospatial processing. *The rostral middle frontal gyrus* - the fourth most informative region - is associated with working memory and visual attention.

DSST score in patients with dementia. *The inferior parietal gyrus* is the highest ranking predictor in the model of DSST scores in the demented patients.

5.2.5 Structural predictors of TMT score

TMT is a neuropsychological test that reflects visuospatial abilities, information processing speed, sustained attention, motor speed, working and rote memory. It shows the time spent on taking the test. *Choroid plexus* volume ranks first in forecasting TMT scores in the cognitively preserved population (see Figure 7). Since the role of this structure in cognition has not been fully described, it's challenging to identify the tasks that are affected by structural alterations to the choroid plexus. The volume of *WM hypointensities* takes second place as a TMT performance predictor. Since the WM lesions reduce motor and processing speed, impair memory, visuospatial function and attention, it's plausible that they rank high in prognosing the test scores. *The middle temporal lobe* is the next valuable structure in the list. It is involved in working memory, attention and visuospatial perception. *The lateral occipital cortex* is a neural center for visual recognition. It also has a high predictive value for the TMT scores.

TMT score in patients with MCI. In the MCI group, *the inferior parietal cortex* is the strongest predictor because of its role in working memory, attention, visuospatial processing, and motor speed. The second best predictor for TMT scores is *the superior marginal cortex* which is involved in information processing.

TMT score in patients with dementia. Finally, the *inferior parietal cortex* and the *grey matter* outperform all other analysed brain regions in detecting TMT scores among the demented patients.

REFERENCES

1 .Cavanna AE, Trimble MR. The precuneus: a review of its functional anatomy and behavioural correlates. *Brain* **129** (2006) 564–583.

2 .Kumral E, Bayam FE, Özdemir HN. Cognitive and behavioral disorders in patients with precuneal infarcts. *European Neurology* **84** (2021) 157–167.

3 .Ruitenberg A, Den Heijer T, Bakker SL, Van Swieten JC, Koudstaal PJ, Hofman A, et al. Cerebral hypoperfusion and clinical onset of dementia: the rotterdam study. *Annals of Neurology: Official Journal of the American Neurological Association and the Child Neurology Society* **57** (2005) 789–794.

4 .Liu XJ, Che P, Xing M, Tian XB, Gao C, Li X, et al. Cerebral hemodynamics and carotid atherosclerosis in patients with subcortical ischemic vascular dementia. *Frontiers in aging neuroscience* **13** (2021).

5 .Coutu JP, Goldblatt A, Rosas HD, Salat DH, (ADNI ADNI, et al. White matter changes are associated with ventricular expansion in aging, mild cognitive impairment, and alzheimer's disease. *Journal of Alzheimer's Disease* **49** (2016) 329–342.

6 .Haller S, Kövari E, Herrmann FR, Cuvinciuc V, Tomm AM, Zulian GB, et al. Do brain t2/flair white matter hyperintensities correspond to myelin loss in normal aging? a radiologic-neuropathologic correlation study. *Acta neuropathologica communications* **1** (2013) 1–7.

7 .Statsenko Y, Habuza T, Smetanina D, Simiyu GL, Uzianbaeva L, Neidl-Van Gorkom K, et al. Brain morphometry and cognitive performance in normal brain aging: Age-and sex-related structural and functional changes. *Frontiers in Aging Neuroscience* (2022).

8 .Min J, Moon WJ, Jeon JY, Choi JW, Moon YS, Han SH. Diagnostic efficacy of structural mri in patients with mild-to-moderate alzheimer disease: automated volumetric assessment versus visual assessment. *American Journal of Roentgenology* **208** (2017) 617–623.

9 .Nordahl CW, Ranganath C, Yonelinas AP, DeCarli C, Fletcher E, Jagust WJ. White matter changes compromise prefrontal cortex function in healthy elderly individuals. *Journal of cognitive neuroscience* **18** (2006) 418–429.

10 .Lockhart SN, Mayda AB, Roach AE, Fletcher E, Carmichael O, Maillard P, et al. Episodic memory function is associated with multiple measures of white matter integrity in cognitive aging. *Frontiers in human neuroscience* **6** (2012) 56.

11 .Fornari LHT, da Silva Júnior N, Carpenedo CM, Hilbig A, de Mello Rieder CR. Striatal dopamine correlates to memory and attention in parkinson's disease. *American Journal of Nuclear Medicine and Molecular Imaging* **11** (2021) 10.

12 .Venkatraman VK, Aizenstein H, Guralnik J, Newman AB, Glynn NW, Taylor C, et al. Executive control function, brain activation and white matter hyperintensities in older adults. *Neuroimage* **49** (2010) 3436–3442.

13 .Rizvi B, Narkhede A, Last BS, Budge M, Tosto G, Manly JJ, et al. The effect of white matter hyperintensities on cognition is mediated by cortical atrophy. *Neurobiology of aging* **64** (2018) 25–32.

14 .Ruiz NA, Meager MR, Agarwal S, Aly M. The medial temporal lobe is critical for spatial relational perception. *Journal of Cognitive Neuroscience* **32** (2020) 1780–1795.

15 .Botzung A, Philippi N, Noblet V, Loureiro de Sousa P, Blanc F. Pay attention to the basal ganglia: a volumetric study in early dementia with lewy bodies. *Alzheimer's Research & Therapy* **11** (2019) 1–9.

16 .Alisch JS, Kiely M, Triebswetter C, Alsameen MH, Gong Z, Khattar N, et al. Characterization of age-related differences in the human choroid plexus volume, microstructural integrity, and blood perfusion using multiparameter magnetic resonance imaging. *Frontiers in Aging Neuroscience* (2021) 613.

17 .Lizano P, Lutz O, Ling G, Lee AM, Eum S, Bishop JR, et al. Association of choroid plexus enlargement with cognitive, inflammatory, and structural phenotypes across the psychosis spectrum. *American Journal of Psychiatry* **176** (2019) 564–572.

18 .Kratzer I, Ek J, Stolp H. The molecular anatomy and functions of the choroid plexus in healthy and diseased brain. *Biochimica et Biophysica Acta (BBA)-Biomembranes* **1862** (2020) 183430.
